# In Vivo Cell Fusion between Mesenchymal Stroma/Stem-Like Cells and Breast Cancer Cells

**DOI:** 10.3390/cancers11020185

**Published:** 2019-02-05

**Authors:** Catharina Melzer, Juliane von der Ohe, Ralf Hass

**Affiliations:** Biochemistry and Tumor Biology Lab, Department of Obstetrics and Gynecology, Hannover Medical School, D-30625 Hannover, Germany; melzer.catharina@mh-hannover.de (C.M.); Ohe.Juliane.von.der@mh-hannover.de (J.v.d.O.)

**Keywords:** in vivo fusion, cancer hybrid cells, mesenchymal stem cells, breast cancer, tumor microenvironment

## Abstract

Cellular communication within the tumor microenvironment enables important interactions between cancer cells and recruited adjacent populations including mesenchymal stroma/stem-like cells (MSC). These interactions were monitored in vivo following co-injection of GFP-labeled human MSC together with mcherry-labeled MDA-MB-231 breast cancer cells in NODscid mice. Within 14 days of tumor development the number of initially co-injected MSC had significantly declined and spontaneous formation of breast cancer/MSC hybrid cells was observed by the appearance of double fluorescing cells. This in vivo fusion displayed a rare event and occurred in less than 0.5% of the tumor cell population. Similar findings were observed in a parallel in vitro co-culture. Characterization of the new cell fusion products obtained after two consecutive flow cytometry cell sorting and single cell cloning revealed two populations, termed MDA-hyb3 and MDA-hyb4. The breast cancer fusion cells expressed both, GFP and mcherry and displayed more characteristics of the MDA-MB-231 cells than of the parental MSC. While little if any differences were determined in the proliferative capacity, a significant delay of MDA-hyb3 cells in tumor formation was observed when compared to the parental MDA-MB-231 cells. Moreover, MDA-hyb3 cells developed an altered pattern of distant organ metastases. These findings demonstrated dynamic tumor changes by in vivo and in vitro fusion with the development of new breast cancer hybrid cells carrying altered tumorigenic properties. Consequently, cancer cell fusion contributes to progressively increasing tumor heterogeneity which complicates a therapeutic regimen.

## 1. Introduction

Cell fusion in general represents a rare biological process which requires close proximity between fusogenic cell partners and is tightly regulated by multiple pathways which, however, are not yet fully understood [1]. This process of hybrid cell formation besides entosis or cell cannibalism is associated with a proper alignment of certain glycoproteins and a permissive lipid composition of the involved parts of the cell membranes to facilitate the initiation of a fusion event. Moreover, an acidified and hypoxic microenvironment detectable in several tumors can act as fusogenic triggers for aberrant spontaneous cell fusion or a so-called process of “accidental cell fusion” [2,3]. Indeed, previous work has demonstrated that hypoxia-induced apoptosis stimulates fusion between breast cancer cells and mesenchymal stroma/stem-like cells whereby the newly formed hybrids demonstrated enhanced migratory capacity [4]. Physiologically observed cell fusions include the generation of muscle fibers by a continuous homo- or autofusion of myoblasts to form multi-nucleated myocytes. Moreover, autofusion of osteoclasts contributes to bone regeneration and metabolic activity. Furthermore, fusion of fetal trophoblasts creates syncytiotrophoblasts which are involved in placenta formation. In addition, heterofusion between egg and sperm combines their haploid gamete genomes and forms a diploid embryonic stem cell [5,6].

Cell fusion also occurs within the tumor microenvironment between cancer cells and neighboring non-malignant cells including mesenchymal stroma/stem-like cells (MSC) [7,8]. These stroma/stem-like cells represent a heterogeneous population preferentially residing in perivascular niches of nearly all kinds of human tissues [9,10]. Despite biological differences according to their tissue-specific origins, MSC share distinct surface protein expressions such as CD73, CD90, and CD105, and they exhibit the capability to differentiate at least along certain phenotypes of the mesodermal lineage [11,12,13,14]. Physiological functions of MSC include among others the regulation of stem cell homeostasis in the bone marrow and an accumulation at damaged or injured tissues to utilize repair processes [15], support angiogenesis [16], and modulate immune cell functions [17], preferably also at tumor sites.

Interaction between cancer cells and MSC in the tumor microenvironment represents a complex multistep cascade whereby an accidental merger, e.g., between breast cancer cells and MSC as a rare event, can be observed within less than five minutes [18]. This is associated with the generation of cancer hybrid cells displaying new cancer cell populations with altered biological properties [19,20].

The molecular mechanisms of cell fusion processes include a reorganization of the actin cytoskeleton by different adhesion molecules to generate membrane structures with appropriate accumulation of transmembrane fusogenic proteins [21,22]. Focal membrane protrusions containing lamellipodia and/or filopodia allow the two adjacent cell membranes to localize in close proximity, whereby contacts create microdomains that favor exchange of membrane patches and cytosolic parts between the attached cells. Moreover, certain cell type-specific fusogenic proteins are required, e.g., syncytin-1 and -2 which originate as truncated viral genes such as the HERV-W retroviral envelope genes [23] and are predominantly detectable in syncytiotrophoblasts of placenta tissue but also in certain solid tumors [18]. Previous findings have demonstrated that heterofusion of breast cancer cells with endothelial cells involves endothelial cell associated ASCT2 (alanine, serine, and cysteine selective transporter-2) which functions as a syncytin receptor present on breast cancer cells [24].

However, the underlying regulatory interplay and physiological relevance for hybrid cell formation by spontaneous breast cancer cell fusion with MSC remains unclear. The present work extends the existing knowledge about in vitro data by providing evidence for breast cancer/MSC fusion in vivo. Moreover, newly generated hybrid cell populations from previous fusion processes between human breast cancer cells and MSC are characterized.

## 2. Results

**Figure 1:**
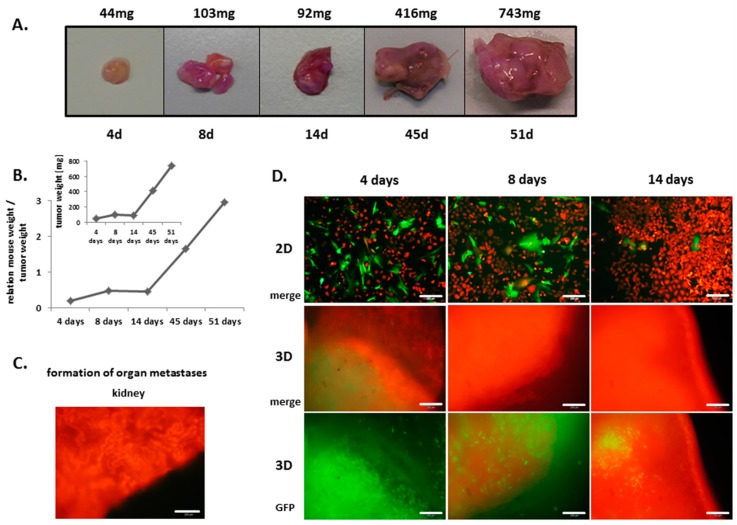
(**A**) NOD/scid mouse tumors were derived following subcutaneous co-injection of 2 × 10^6^ human MSC300415^GFP^ with 2 × 10^6^ MDA-MB-231^cherry^ breast cancer cells for different time points (4 days, 8 days, 14 days, 45 days, and 51 days). (**B**) Ratio of mouse weight to tumor weight was compared within different time points. The diagram insert represents tumor weight only. (**C**) Formation of distant metastases was evaluated in mice after 45 days and 51 days of tumor development by investigation of cherry- and green fluorescent protein (GFP)-fluorescence in organs. One exemplary picture from kidney metastasis is documented. Bars represent 200 µm. (**D**) Morphology and cell ratio of 2D co-cultures between MSC300415^GFP^ and MDA-MB-231^cherry^ was compared to appropriate 3D tumor tissues after 4 days, 8 days, and 14 days as documented by GFP fluorescence microscopy and by a merge of mcherry and GFP fluorescence (merge). Particularly the 3D fluorescence micrographs (merge) after 8 days and 14 days demonstrate a diffuse red fluorescence within the tumor tissue indicating a predominant majority of breast cancer cells. Bars represent 200 µm.

In vivo co-injection of MSC300415^GFP^ with MDA-MB-231^cherry^ demonstrated tumor development already after 4 days. In parallel, the initial tumor tissue color changed to a red phenotype after 8 days indicating a predominant accumulation of MDA-MB-231^cherry^ cells (Figure 1A). The tumor weight of 44 mg after 4 days increased to 743 mg after 51 days post injection whereby mouse weight increased from initially 20 g to 25 g after 51 days (Figure 1A,B). Distant organ metastases in liver, heart, lung, spleen, or brain remained undetectable whereas metastasized mcherry-positive fluorescing cancer cells exclusively appeared in kidney sections of the two mice after 45 and 51 days post injection (Figure 1C). Of interest, these metastases demonstrated only mcherry fluorescence without any traceable GFP fluorescence. This is also supported by fluorescence microscopy demonstrating that in parallel to the increasing tumor development, the amount of detectable MSC^GFP^ decreased over time (Figure 1D). Indeed, after initiating the 2D in vitro co-culture and the 3D in vivo co-injection with both cell populations in equal amounts, fluorescence microscopy images of tumor tissues from mice sacrificed after 4, 8, and 14 days clearly documented the decline of cells expressing GFP fluorescence (Figure 1D, middle and lower panel). Concomitantly, the amount of cherry fluorescence continuously increased over time. These in vivo observations were substantiated by 2D in vitro co-cultures with decreasing numbers of GFP-expressing MSC (Figure 1D upper panel). 

**Figure 2:**
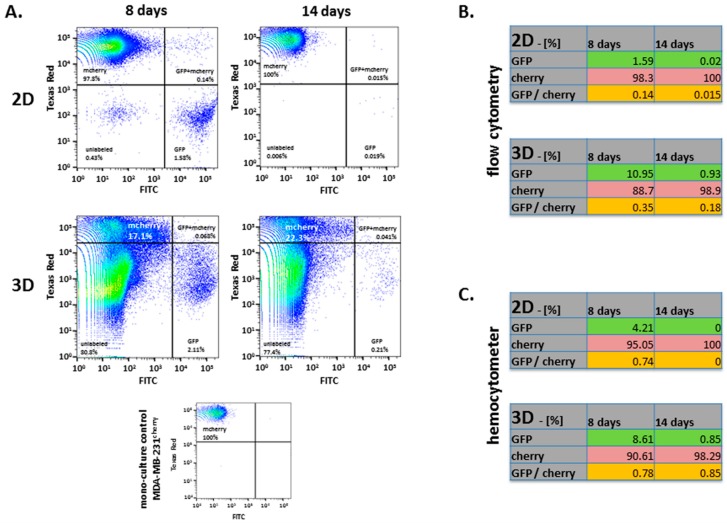
Analysis of GFP, cherry and GFP+cherry fluorescence in 8 and 14 days 2D cultures and ex vivo 3D tumors, including an MDA-MB-231^cherry^ mono-culture control, were (**A**) examined by flow cytometric analysis and (**B**) the proportions were adjusted to 100% after exclusion of unlabeled cell populations. (**C**) In addition, percentages of GFP and cherry fluorescence were quantified by cell counting in a hemocytometer. All results were compared to the appropriate 2D co-cultures on plastic dishes (Greiner Bio-One, Kremsmünster, Austria).

Following trysinization of the 2D co-cultures and dissociation of 3D tumor tissues after 8 and 14 days, respectively, the collected cell populations were quantified by flow cytometry for mcherry, GFP and double fluorescing cells whereby all non-fluorescing cells were excluded as negative populations (Figure 2A). From 100% of the fluorescing populations which initially equals 50% of mcherry and GFP cells, the amount of GFP-positive cells within the tumor tissue declined to about 11% after 8 days and further dropped to 0.93% after 14 days (Figure 2B, lower panel). In contrast, the percentage of mcherry-positive cells increased from 88.7% after 8 days up to 98.9% after 14 days. A small population of dual fluorescent cells was detectable at both time points (0.35% at 8 days and 0.18% at 14 days) (Figure 2B, lower panel). Even more stringent effects were observed in the corresponding 2D co-cultures. About 1.6% of GFP-positive cells remained detectable after 8 days of co-culture and were largely overgrown by the mcherry breast cancer cells with a remaining population of 0.02% after 14 days. Similarly, the amount of double positive hybrid cells dropped from 0.14% after 8 days to about 0.015% after 14 days (Figure 2B, upper panel).

Similar findings were obtained in an independent cell counting evaluation of both, previously co-injected 3D tumor tissue-dissociated cell populations and 2D co-cultures by using a hemocytometer (Figure 2C).

**Figure 3:**
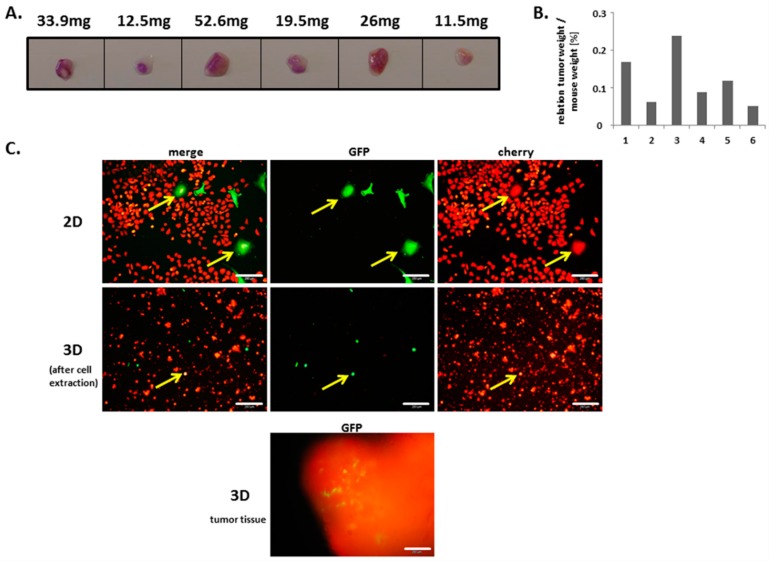
(**A**) In vivo formation of hybrid cells during tumor development was tested after subcutaneous co-injection of 10^6^ human MSC290115^GFP^ together with 10^6^ MDA-MB-231^cherry^ breast cancer cells in NOD/scid mice for 14 days. (**B**) Ratio of mouse weight to tumor weight was compared between 6 tumors from 3 NOD/scid mice. (**C**) Formation of hybrid cells as indicated by yellow arrows was detectable by fluorescence microscopy in both, 2D in vitro co-cultures (upper panel) and in 3D ex vivo tumor tissues following extracellular matrix digestion and cell isolation (middle panel). In vivo presence of MSC290115^GFP^ in tumor tissues after 14 days is exemplarily documented (lower micrograph). Bars represent 200 µm.

In a further in vivo approach, tumor development and potential in vivo cell fusion were examined following subcutaneous co-injection of MDA-MB-231^cherry^ breast cancer cells with MSC from a different donor (MSC290115) into both flanks of 3 NOD/scid mice. The mice were sacrificed after 14 days post injection and tumor tissues were documented and weighed (Figure 3A). Differences in tumor size compared to the data in Figure 1A after 14 days resulted from the reduced initial number of co-injected cells. Calculations of the relationship of tumor weight to mouse weight revealed a distribution between 0.05% and 0.24% (Figure 3B). Following dissociation of all six tumor tissues, the appearance of dual fluorescent cells was documented by fluorescence microscopy in one dissociated representative tumor population (Figure 3C middle panel, yellow arrows). Appropriate 2D co-cultures were performed in parallel and likewise demonstrated the presence of double fluorescing hybrid cells (Figure 3C upper panel, yellow arrows). Control fluorescence pictures of tumor tissue thin sections revealed the presence of both, mcherry-labeled breast cancer cells and GFP-expressing MSC (Figure 3C lower panel).

**Figure 4:**
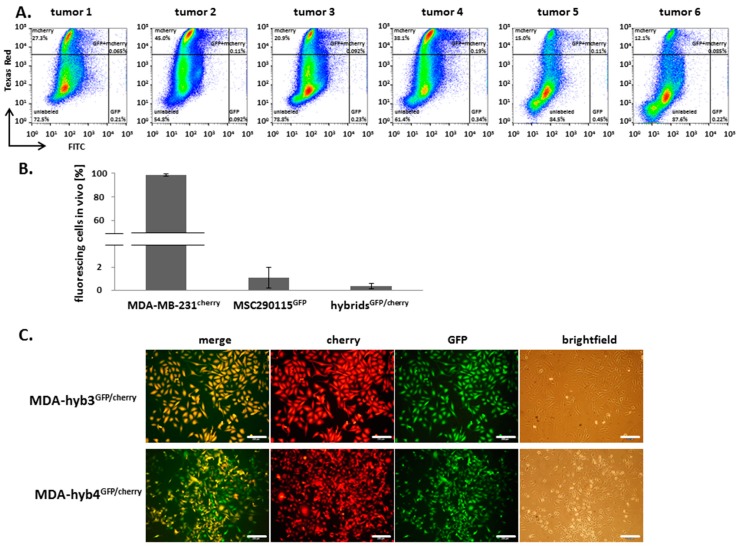
After dissociation of 6 in vivo tumor tissues, supernatants were analyzed by flow cytometry (**A**) and quantified for MDA-MB-231^cherry^, MSC290115^GFP^ and hybrid cells^GFP/cherry^ fluorescence. Data represent the mean ± S.D. from all 6 tumor tissue supernatants (**B**). Hybrid cell formation was observed in 2D co-cultures between MSC290115^GFP^ and MDA-MB-231^cherry^ resulting in GFP and cherry positive yellow fluorescing cells. Separation of hybrid cells was performed by fluorescence-activated cell sorting (FACS) and subsequent single cell cloning. Two different clones (MDA-hyb3 and MDA-hyb4) were isolated (**C**). Bars represent 200 µm.

Isolated cells from dissociated tumor tissues were further examined by flow cytometry for the existence of dual fluorescent fusion cells after co-injection. Indeed, in vivo fusion cells were confirmed in the fluorescence-activated cell sorter (FACS) dot plots (Figure 4A). While most cells were unlabeled, the vast majority of fluorescing cells was represented by the mcherry breast cancer population. Still, MSC^GFP^ and in vivo fusion cells carrying mcherry and GFP double fluorescence displayed a detectable population in all tumor tissues (Figure 4A). Quantification of the fluorescing cells from FACS analysis revealed about 98% of mcherry breast cancer cells after 14 days in vivo (Figure 4B). Conversely, the amount of MSC^GFP^ decreased from initially 50% to almost 1% after 14 days. At the same time formation of GFP and cherry positive breast cancer fusion cells acquired 0.41% (Figure 4B).

While corresponding 2D co-cultures revealed a similarly detectable amount of hybrid cells, repeated fluorescence activated cell sorting (FACS) was performed after 7 days co-culture with subsequent single cell cloning for dual fluorescent cells. Following culture of 5473 double-sorted dual fluorescing cell clones, two different proliferating hybrid clones were selected termed MDA-hyb3^GFP/cherry^ and MDA-hyb4^GFP/cherry^ demonstrating different morphologies and expression of GFP and cherry fluorescence (Figure 4C). These findings demonstrated that MSC–cancer cell fusion represents a rare event whereby the initially resulting hybrid cells underwent a substantial post-fusion selection process.

**Figure 5:**
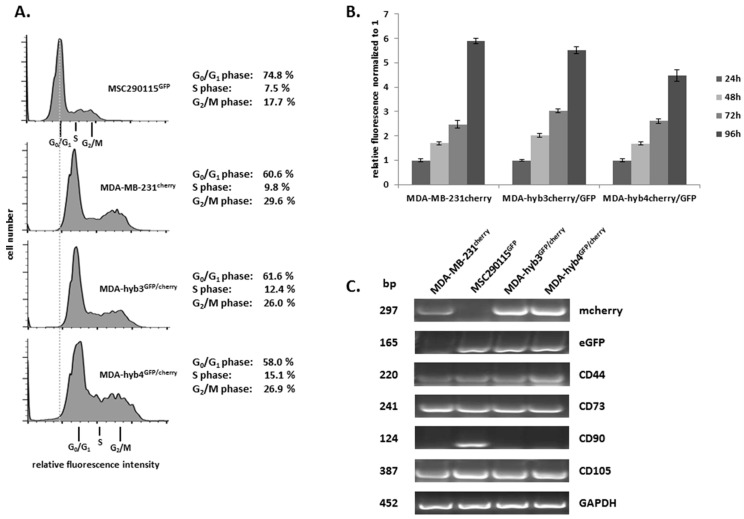
(**A**) Cell cycle analysis of MDA-hyb3 and MDA-hyb4 in contrast to the parental cell populations revealed hyperdiploidy of both hybrid cell populations. (**B**) Proliferation of hybrid cell lines was examined in a fluoroskan assay for 24 h up to 96 h and compared to proliferation capacity of the parental MDA-MB-231^cherry^ breast cancer cells. Data represent the mean ± S.D. (*n* = 10) whereby fluorescence values after 24 h were set to 1. (**C**) PCR analysis was performed for mcherry, eGFP and MSC stem-like markers CD44, CD73, CD90 and CD105. Expression of parental MDA-MB-231^cherry^ and MSC290115^GFP^ populations were compared to the two hybrid populations. Expression levels of GAPDH served as control.

Cell cycle analysis of these two hybrid populations demonstrated a similar cell cycle distribution as compared to MDA-MB-231^cherry^ breast cancer cells. While the parental MSC290115^GFP^ displayed a cell cycle with normal diploid DNA content and 17.7% cells in the G2/M phase, the pronounced cell cycle shift towards an elevated fluorescence intensity and accordingly, increased DNA content suggests aneuploidy/hyperdiploidy of MDA-hyb3 and MDA-hyb4, respectively, like the mixed MDA-MB-231^cherry^ karyotype carrying 52 to 68 chromosomes (Figure 5A, dotted line). In addition, MDA-MB-231^cherry^ and the two hybrid populations exhibited G2/M cells between 26% and 29% suggesting enhanced cell cycle progression as compared to the parental MSC (Figure 5A). 

The proliferation rate assessed by fluoroskan assay revealed little if any differences of MDA-hyb3 in comparison to the parental MDA-MB-231^cherry^ cells while the proliferative potential of MDA-hyb4 was slightly decreased after 24 h up to 96 h (Figure 5B).

RT-PCR analysis substantiated hybrid cell formation of MDA-hyb3 and MDA-hyb4 by simultaneous expression of both fluorescence genes mcherry and GFP whereby exclusive expression of mcherry was detectable in MDA-MB-231^cherry^ and eGFP in MSC290115^GFP^ (Figure 5C). Although mRNA transcript levels of the MSC-related stemness marker CD44, CD73, and CD105 were expressed in all four cell populations, CD90 expression remained limited to MSC^GFP^ further supporting a reduced MSC-like phenotype of the two hybrid populations MDA-hyb3 and MDA-hyb4.

Together, these data suggested the isolation of two new cell populations after spontaneous fusion of MSC290115^GFP^ with MDA-MB-231^cherry^ with a congruous proliferative capacity and cell cycle pattern as compared to the parental MDA-MB-231^cherry^.

**Figure 6:**
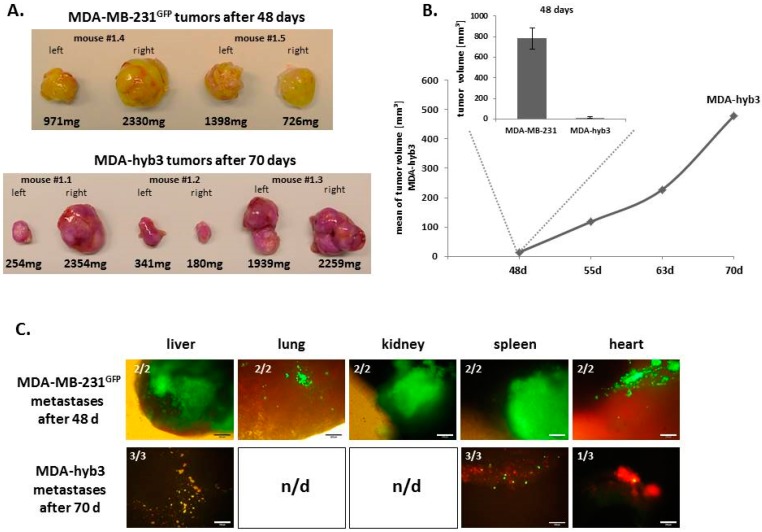
(**A**) MDA-MB-231^GFP^ cells-induced tumors in both flanks of two NODscid mice were harvested after 48 days whereas MDA-hyb3-induced tumors from three mice were collected after 70 days displaying a similar average tumor size. (**B**) Progressively increasing tumor volumes of MDA-hyb3-induced tumors were monitored and evaluated from 48 days to 70 days when the tumor volume reached an average size of that observed for parental MDA-MB-231^GFP^ cells after 48 days (inserted bar diagram). (**C**) Formation and quantification of distant organ metastases in representative fluorescence pictures is demonstrated for MDA-MB-231^GFP^ cells after 48 days as compared to MDA-hyb3-mediated metastases after 70 days. n/d = not detectable. Bars represent 200 µm.

According to the similar proliferation rate of MDA-hyb3 and MDA-MB-231, these cell populations were compared for their capability to develop in vivo tumors and potential organ metastases in NODscid mice (Figure 6). While MDA-MB-231^GFP^ cells promoted subcutaneous tumors with an average weight of 1356 mg within 48 days, this tumor development was significantly delayed in MDA-hyb3-induced tumors reaching an average weight of 1221 mg after 70 days (Figure 6A). Likewise, the MDA-MB-231^GFP^ cell-associated tumor volume of about 781 mm^3^ was paralleled by a tumor volume of 14 mm^3^ in MDA-hyb3-induced tumors after 48 days (Figure 6B, inserted bar diagram). Thereafter, the MDA-hyb3 tumors progressively increased to an average volume of 478 mm^3^ after 70 days (Figure 6B). Distant organ metastases were detectable in all investigated organs in MDA-MB-231^GFP^-induced tumors after 48 days. In contrast, double fluorescing cells of MDA-hyb3 remained undetectable in lung and kidney after 70 days. Moreover, metastatic cells in the heart were identified only in one out of three MDA-hyb3 tumor mice (Figure 6C). Together, these data indicated a retarded tumor development with reduced formation of metastases in MDA-hyb3 cells when compared to the parental MDA-MB-231^GFP^ cells.

## 3. Discussion

A variety of mechanisms contribute to indirect interaction of breast cancer cells with MSC including the release of soluble factors (cytokines, chemokines, enzymes, metabolites), microvesicles and exosomes, which can induce among others cancer cell alteration and a retrodifferentiation program for potential formation of cancer stem-like cells [25,26]. Moreover, interaction of breast cancer cells with populations of perivascular regions such as pericytes and MSC can also contribute to tumor cell dormancy [27]. Furthermore, during indirect interaction with ovarian cancer cells, human MSC were suggested to promote tumor growth and support proliferation and survival [28]. Indeed, co-culture of different breast and ovarian carcinoma cells with MSC is associated both, in vitro and in vivo with progressive reduction of MSC by overgrowing cancer cells while intercellular communication of MSC with these cancer cells promotes mutual functional alteration including enhanced tumor growth and elevated metastatic potential [20,29].

Alternatively, more close and direct interaction of cancer cells with adjacent neighboring cell populations such as immune cells, adipose tissue cells, endothelial cells or local tissue-associated MSC involve nanotube formation, trogocytosis, or cell fusion including cannibalism or entosis. Spontaneous or accidental cell fusion between neoplastic breast epithelial cells and MSC represents a rare event while the fusion process itself occurs rapidly within a few minutes [18]. Successful cell fusion with the generation of viable hybrid cells requires a coordinated chromosomal restructure and a coherent nuclear rearrangement and reprogramming [30,31]. Otherwise, cell fusion and associated post-fusion selection processes can generate aneuploidy, chromosomal instability, and DNA damage, all of which cause multiple genetic changes including genetic aberrations and a potentially neoplastic development [6,32]. Indeed, previous work suggested that natural progression of cancer is accompanied by cell fusion between cancer cells and non-tumorigenic adjacent cells leading to massive genomic alterations and enhanced clonal diversity, thus, increasing tumor heterogeneity [33]. Consequently, detection of mcherry and GFP double fluorescing breast cancer fusion cells in vivo add to the tumor heterogeneity and may contribute to facilitated development of distant tissue metastases which is also supported by the findings of Chitwood et al. [34].

The generation of new cancer hybrid populations in breast cancer [4,18,19,34] and ovarian cancer [35] was documented by cellular fusion with interacting MSC. In addition, previous work has demonstrated that spontaneous fusion of certain human breast epithelial with breast cancer cells generated five different hybrid populations exhibiting cancer stem cell-like properties [36]. While reports on several tumors suggest hybrid cell generation by acquisition of certain donor cell genomes, evidence for in vivo cancer cell fusion using transplantable cancer cell lines represents a largely simplified model which limits translational clinical aspects [37]. Moreover, clonal convergence after post-fusion selection provides only a narrowed window of the hybrid populations. Thus, fusion of cancer cells with leukocytes, particularly macrophages could generate hybrid cells with altered metastatic dissemination contributing to enhanced tumor heterogeneity [37,38].

The use of metastasis-forming breast cancer cells as fusion partner in this study revealed alterations in the metastatic capacity of the new cancer hybrid cells. Although distinct MSC-typical stem cell markers are also acquired during heterotypic fusion of breast or ovarian cancer cells with MSC, the resulting hybrid populations displayed altered or reduced tumorigenic potential. While recent studies demonstrated enhanced tumor growth and elevated formation of metastases in fusion populations of MDA-MB-231 breast cancer with human MSC051212, termed MDA-hyb1 and MDA-hyb2, a more favorable property acquired by these tumor hybrid populations was an increased sensitivity to various chemotherapeutic compounds in contrast to their parental breast cancer counterparts [19]. The present newly established MDA-hyb3 and MDA-hyb4 breast cancer hybrid populations were derived after fusion with MSC from a different donor (MSC290115) whereby MDA-hyb3 cells displayed similar proliferative potential in vitro but retarded tumor development and reduced metastatic potential in vivo unlike the parental MDA-MB-231 cells. These findings indicate overall increased tumor heterogeneity, however, the involvement of MSC in these fusion processes reflects certain physiologically beneficial contributions e.g. by elevated drug response [39] and/or reduced tumorigenicity of the cancer hybrid cells. Indeed, this conclusion is also supported by in vivo data obtained with the SK-OV-3/MSC fused ovarian cancer hybrid populations SK-hyb1 and SK-hyb2 demonstrating that the ovarian cancer fusion cells had completely lost the capacity of tumor formation [35].

In summary, the present work demonstrates that cancer cell fusion can occur in vivo by breast cancer hybrid cell formation in NODscid mice which extends the previous findings of in vitro fusion [19]. Moreover, the characterization of the newly generated MDA-hyb3 and MDA-hyb4 populations broadens tumor heterogeneity by exhibiting reduced tumorigenic properties as compared to their parental counterpart or to the previously described more aggressive MDA-hyb1 and MDA-hyb2 breast cancer hybrids [19]. While regulatory orchestration of cancer cell fusion processes is far from being understood, further evaluation of the heterogenic functionality of cancer hybrid cells is required with respect to enhanced or reduced tumorigenic potential and their role in the production of metastatic lesions or the expression of stem-like characteristics among others.

## 4. Materials and Methods

### 4.1. Cell Culture

Primary human MSC were isolated from umbilical cord tissue as described previously [40]. MSC were cultured at 37 °C with 5% CO_2_ in αMEM (Sigma Chemie GmbH, Steinheim, Germany) supplemented with 10% of allogeneic human AB-serum (blood from 31 male AB donors was commercially obtained from a blood bank, Hannover Medical School, Germany, and processed to serum), 100 U/mL penicillin, 100 µg/mL streptomycin, and 2 mM l-glutamine (Sigma). The study was conducted in accordance with the Declaration of Helsinki, and the Ethics Committee of the Hannover Medical School approved the isolation and culturing of primary human MSC, Project #443 on 26 February 2009, and informed written consent was obtained from each patient.

Subculture of MSC was performed by accutase (Sigma) treatment for 3 min at 37 °C. For the experiments, MSC from two different donors (MSC290115 and MSC300415) were applied.

Human breast carcinoma cell line MDA-MB-231 was commercially obtained from ATCC, Manassas, VA, USA. This breast cancer cell line was cultured in Leibovitz’s L-15-medium (Life technologies, Darmstadt, Germany) supplemented with 10% (*v*/*v*) FCS, 100 U/mL penicillin, 100 µg/mL streptomycin, and 2 mM l-glutamine (Sigma). Subculture was performed by trypsin/EDTA (Biochrom GmbH, Berlin, Germany) treatment for 5 min at 37 °C.

Cells were tested for mycoplasma by the luminometric MycoAlert Plus mycoplasma detection kit (Lonza Inc., Rockland, ME, USA) according to the manufacturer’s recommendations. Cell line authentication was performed by short tandem repeat (STR) fragment analysis using the GenomeLab human STR primer set (Beckman Coulter Inc., Fullerton, CA, USA). For fluorescence labeling and discrimination of the cell populations in co-culture, MSC and the cancer cells were transduced with a 3rd generation lentiviral SIN vector containing the eGFP and the mcherry gene, respectively, as indicated in previous work [20].

### 4.2. In Vivo Experiments

Animal experiments using NOD/scid mice were approved by the institutional licensing committee reference # 33.19-42502-04-15/1992 on 18 December 2015 and were performed by obeying the internationally recognized guidelines on animal welfare.

In a first set of in vivo experiments, 2 × 10^6^ GFP-labeled MSC300415^GFP^ together with 2 × 10^6^ cherry-labeled MDA-MB-231^cherry^ were co-injected subcutaneously into 5 female NOD/scid mice (5 to 6 weeks old), respectively. After 4 days post injection, all 5 mice had developed subcutaneous tumors. Mice were sacrificed by cervical dislocation at different time points, 4, 8, 14, 45, and 51 days post injection. Tumors developed after 8 and 14 days were dissected, washed in PBS and incubated in tumor dissociation buffer (a mixture of 50% (*v*/*v*) reconstituted tumor dissociation enzyme reagent (DCS Innovative Diagnostik-Systeme GmbH, Hamburg, Germany) and 50% (*v*/*v*) serumfree medium) for 5 h at 37 °C. Liberated cells from digested tumor tissue in the supernatant were harvested by centrifugation (360 *g*/5 min), counted in a hemocytometer, and analyzed by flow cytometry. 

In parallel to the in vivo study, a similar 2D in vitro co-culture of MSC300415^GFP^ and MDA-MB-231^cherry^ (same cell ratio) was performed for 8 and 14 days, respectively. Organs from mice sacrificed after 45 and 51 days were analyzed for metastasis by fluorescence microscopy. 

In a second set of in vivo experiments, 1 × 10^6^ MSC290115^GFP^ together with 1 × 10^6^ MDA-MB-231^cherry^ cells were co-injected subcutaneously into the left and the right flank of 3 female NOD/scid mice (5 to 6 weeks old) to minimize the number of animals by generating tumors on both flanks. Although mutual effects from both flanks cannot be ruled out, this does not interfere with the focus of this experimental approach to examine the general possibility of cancer hybrid cell formation in vivo. Half of the cell number from previous in vivo experiment was applied to decrease the velocity of tumor development. After 7 days post injection, all 3 mice had developed subcutaneous tumors on both sides. Mice were sacrificed by cervical dislocation 14 days after cell co-injection. All tumors were dissected, washed in PBS and incubated in tumor dissociation buffer for 5 h at 37 °C. Liberated cells from digested tumor tissue in the supernatant were harvested by centrifugation (360 *g*/5 min), counted in a hemocytometer, documented by fluorescence microscopy, and analyzed by flow cytometry. 

In a third in vivo experiment, a population of 1 × 10^6^ MDA-hyb3^GFP/cherry^ cells was injected subcutaneously into the left and the right flank of 3 female NOD/scid mice (5 to 6 weeks old) and tumor development was compared to 1 × 10^6^ MDA-MB-231^GFP^ similarly injected on both sides of 2 female NOD/scid mice. Tumor progression of MDA-hyb3 tumor was measured using a digital caliper (VWR International) and corresponding tumor volumes (V) were calculated as previously described [41]. Organs from mice sacrificed after 48 days with MDA-MB-231^GFP^ tumors and after 70 days with MDA-hyb3^GFP/cherry^-induced tumors were analyzed for metastasis by fluorescence microscopy.

### 4.3. Flow Cytometric Analysis

For detection and quantification of in vivo-generated hybrid cells, flow cytometry analysis was performed by using the LSRII (BD Bioscience, San Jose, CA, USA) and FlowJo V10 analysis software (BD Bioscience).

### 4.4. Proliferation Measurement by Fluoroskan Assay

Proliferation of MDA-hyb3^GFP/cherry^ and MDA-hyb4^GFP/cherry^ was analyzed by fluoroskan measurements as described previously [42]. Briefly, 1000 cells/well were incubated in flat bottom 96-well plates (Nunc, Thermo Fisher Scientific, Rokslide, Denmark) at appropriate time points for 24 h up to 96 h. After removal of the medium supernatant, cells were lysed with 10% (w/v) SDS and fluorescence was measured by detection of fluorescence intensity of mCherry (excitation: 584 nm, emission: 612 nm) and eGFP (excitation: 485 nm, emission: 520 nm) using the Fluoroskan Ascent FL (Thermo Fisher Scientific, Schwerte, Germany).

### 4.5. Cell Cycle Analysis

Analysis of cell cycle distribution was performed as indicated elsewhere [43]. Briefly, 1 × 10^5^ cells were fixed in 70% ice-cold Ethanol at 4 °C for 24 h. After washing with PBS, fixed cells were stained with 500 µL of DNAse-free RNase (200 U/mL) and 500 µL of propidium-iodide (PI) staining solution (500 µL 0.5 mg/mL propidium iodide in 10mL PBS + 100 µL Trition-X-100) at room temperature protected from light for 30 min and subsequently analyzed by FACScalibur (BD Biosciences) and FlowJo V10 analysis software.

### 4.6. Transcript Analysis by RT-PCR

Total RNA was isolated using RNeasy Mini Kit (Qiagen, Hilden, Germany) according to manufacturer’s instructions. One µg RNA was reverse transcribed into cDNA using 500 µM of dNTP (R0193), 5 µM Oligo(dT)18 primer (S0132), 5 µM Random Hexan primer (S0142), 1 U RiboLockTM RNase Inhibitor (E00381), and 5 U RevertAidTM M-MuLV Reverse Transcriptase (EP0441) in the supplied reaction buffer (all reagents obtained from Thermo Scientific, Schwerte, Germany). The cDNA reactions were performed for 10 min at 25 °C followed by 1 h at 37 °C and stopped at 72 °C for 10 min. cDNA (2.5 µL) was used as a template and amplified by PCR with the following specific primers (customized by Eurofins, MWG GmbH, Ebersberg, Germany) as described previously [19].

## 5. Conclusions

Our findings demonstrate that cell fusion between MSC and breast cancer cells is not limited to a simplified co-culture model in vitro but also displays a rare but evident mechanism of close cellular interaction in vivo. Formation of new cancer hybrid cells by in vivo fusion processes includes the acquisition of new biological properties, thereby contributing to progressively increasing tumor heterogeneity and altered metastatic behavior. Although different cancer hybrid populations can develop distinct tumorigenic and metastatic levels, their diversity may influence clinical outcome and complicates a therapeutic regimen. While these findings also further underscore the biological and clinical relevance of the heterogenic and diverse MSC functionalities during tumor development, molecular signaling mechanisms of cancer cell fusion and corresponding post-fusion selection processes for a clonal convergence to generate certain cancer hybrid populations remain to be elucidated.
